# Protein docking refinement by convex underestimation in the low-dimensional subspace of encounter complexes

**DOI:** 10.1038/s41598-018-23982-3

**Published:** 2018-04-12

**Authors:** Shahrooz Zarbafian, Mohammad Moghadasi, Athar Roshandelpoor, Feng Nan, Keyong Li, Pirooz Vakli, Sandor Vajda, Dima Kozakov, Ioannis Ch. Paschalidis

**Affiliations:** 1 Division of Systems Engineering, Boston University, Boston, Massachusetts United States of America; 2 Department of Mechanical Engineering, Boston University, Boston, Massachusetts United States of America; 3 Department of Biomedical Engineering, Boston University, Boston, Massachusetts United States of America; 4 Department of Electrical and Computer Engineering, Boston University, Boston, Massachusetts United States of America; 5 Department of Applied Mathematics and Statistics and Laufer Center for Physical and Quantitative Biology, Stony Brook University, Stony Brook, New York United States of America; 6 Present Address: 8 Saint Mary’s St., Boston, MA 02215 United States of America

## Abstract

We propose a novel stochastic global optimization algorithm with applications to the refinement stage of protein docking prediction methods. Our approach can process conformations sampled from multiple clusters, each roughly corresponding to a different binding energy funnel. These clusters are obtained using a density-based clustering method. In each cluster, we identify a smooth “permissive” subspace which avoids high-energy barriers and then underestimate the binding energy function using general convex polynomials in this subspace. We use the underestimator to bias sampling towards its global minimum. Sampling and subspace underestimation are repeated several times and the conformations sampled at the last iteration form a refined ensemble. We report computational results on a comprehensive benchmark of 224 protein complexes, establishing that our refined ensemble significantly improves the quality of the conformations of the original set given to the algorithm. We also devise a method to enhance the ensemble from which near-native models are selected.

## Introduction

Proteins are a key element of the cell and play an important role in a variety of cellular functions such as ligand binding, metabolic control, cell signaling and gene regulation. The prediction of the tertiary structure of protein complexes is known as the *protein-protein docking* problem. Several experimental techniques, primarily X-ray crystallography and nuclear magnetic resonance (NMR), are used to predict the 3-dimensional (3D) structure of macromolecular complexes, including proteins, but these methods are usually expensive, time-consuming, and may not be applicable to short-lived molecular complexes. Therefore, computational protein docking methods are very much in need and have attracted considerable attention in the last two decades.

Based on the principles of thermodynamics, the most stable state of a protein complex (the *native* conformation) occurs when its Gibbs free energy attains its minimum value. Binding involves conformational changes to the unbound state of the complex components, affecting their backbones and the side-chains. In this light, the protein docking problem can be posed as an optimization problem in which the variables are the atomic coordinates of the proteins and the objective is to minimize the binding energy of the complex. While this formulation ignores configuration-related entropy terms of free energy, these can be incorporated by post-processing low energy solutions using metrics of density and cluster size.

Despite significant progress in recent years, protein docking is still regarded as a very challenging problem in structural biology due to the complexity of the energy landscape of protein-protein or other protein^1^ interactions. This complexity stems from the fact that the energy function is composed of multiple force-field energy terms (such as the Lennard-Jones potential, solvation, hydrogen bonding, electrostatics, etc.) acting in different space scales and resulting in a multi-frequency behavior of the various energy terms. Therefore, the energy function exhibits multiple deep funnels and extremely many local minima over its multidimensional domain.

In response to this level of complexity, state-of-the-art docking protocols employ a two-stage approach. At the first stage, we use a simplified energy function (expressed as a correlation function) and sample on a conformational space grid an enormous number of docked receptor-ligand conformations, using Fast Fourier Transforms (FFT) for energy evaluation. To conduct this initial sampling we use the protein docking server *ClusPro 2*.*0* which is based on a docking program called PIPER^2^. These conformations are then sorted by their energy values, and the top few thousands with the lowest energy are retained for further processing. At the second stage of docking protocols, we seek to *refine* low energy conformations by moving off-grid and utilizing more elaborate energy functions. Our work in this paper focuses on this *refinement stage*^3^. One of the distinguishing features of our work is that it *does not* assume any prior knowledge about the native structure. In fact, the input to our algorithm is the output of the PIPER docking software, which consists of the lowest energy globally sampled conformations. We evaluate how well these initial conformations are refined by considering the number of good quality solutions in the refined ensemble.

The *refinement problem* we outlined, inherits the complex structure of the binding energy landscape. Approaches that have been considered almost invariably involve efficient sampling and methods that attempt to “smooth” the energy function. A successful strategy is to use *Monte Carlo*-based sampling^4^. An alternative method that resamples around low-energy PIPER structures has also been proposed^5^. A host of methods seek to leverage the *funnel-like* shape of the energy function^6–8^. In fact, similar strategies have been used in protein folding^9–12^. The binding energy funnel is restricted to a neighborhood of the native complex^13^ and there is a free energy gradient toward the native state. However, the funnel is rough, giving rise to many local minima^14^ that correspond to encounter complexes, some of which may be visited along a particular association pathway^15,16^.

## Underestimation

An early algorithm designed for protein folding, the *Convex Global Underestimator* (*CGU)* method^17^, introduced the idea of using an approximation of the envelope spanned by the local minima of the energy function in the form of *convex canonical quadratic* underestimators. CGU, however, used a restricted class of underestimators^18^, limiting its effectiveness. The *Semi-Definite programming-based Underestimation (SDU)* method^18,19^ uses the same approach as CGU but it considers the class of “general” convex quadratic functions to underestimate, in addition to introducing an exploration strategy biased by the underestimator.

In this paper we propose a number of generalizations to SDU. First, and following our earlier preliminary work^20^, we consider the more general class of *convex polynomial functions* for underestimation. Polynomial functions are more flexible than quadratic functions used in the aforementioned methods^17–19^ and can more tightly approximate a funnel.

A second generalization is the ability to handle multiple local funnels in the original cluster presented for refinement. This is important because by deriving a single underestimator (as in Nan *et al*.^20^), we will tend to “average” a complex energy landscape and produce a minimum of the underestimator that may not correspond to a low-energy funnel basin. We resolve this issue by establishing an effective exploration procedure using density-based clustering as follows. First, we run a density-based clustering algorithm on the set of (PIPER) structures which are the inputs to the refinement protocol. This phase eliminates outliers and low-density regions of the conformational space, resulting in multiple sub-clusters whose size is greater than a pre-specified threshold. Then, we construct one underestimator per sub-cluster which allows us to approximate and explore each sub-cluster separately. Finally, we combine all the sampled conformations from all clusters, and pick the low-energy conformations as the output of the refinement protocol.

## Dimensionality Reduction

An important question in underestimation is to determine the appropriate multi-dimensional space in which underestimation takes place. Our experience has suggested that for many complexes, underestimation in the entire 6D space of conformational variables (translations and rotations of the ligand with respect to the receptor) may not be effective and can produce underestimators whose minimum is outside the range of the cluster. This is due to “singularities” of the energy landscape resulting in energy being very steep along some directions and flat along others.

**Figure 1:**
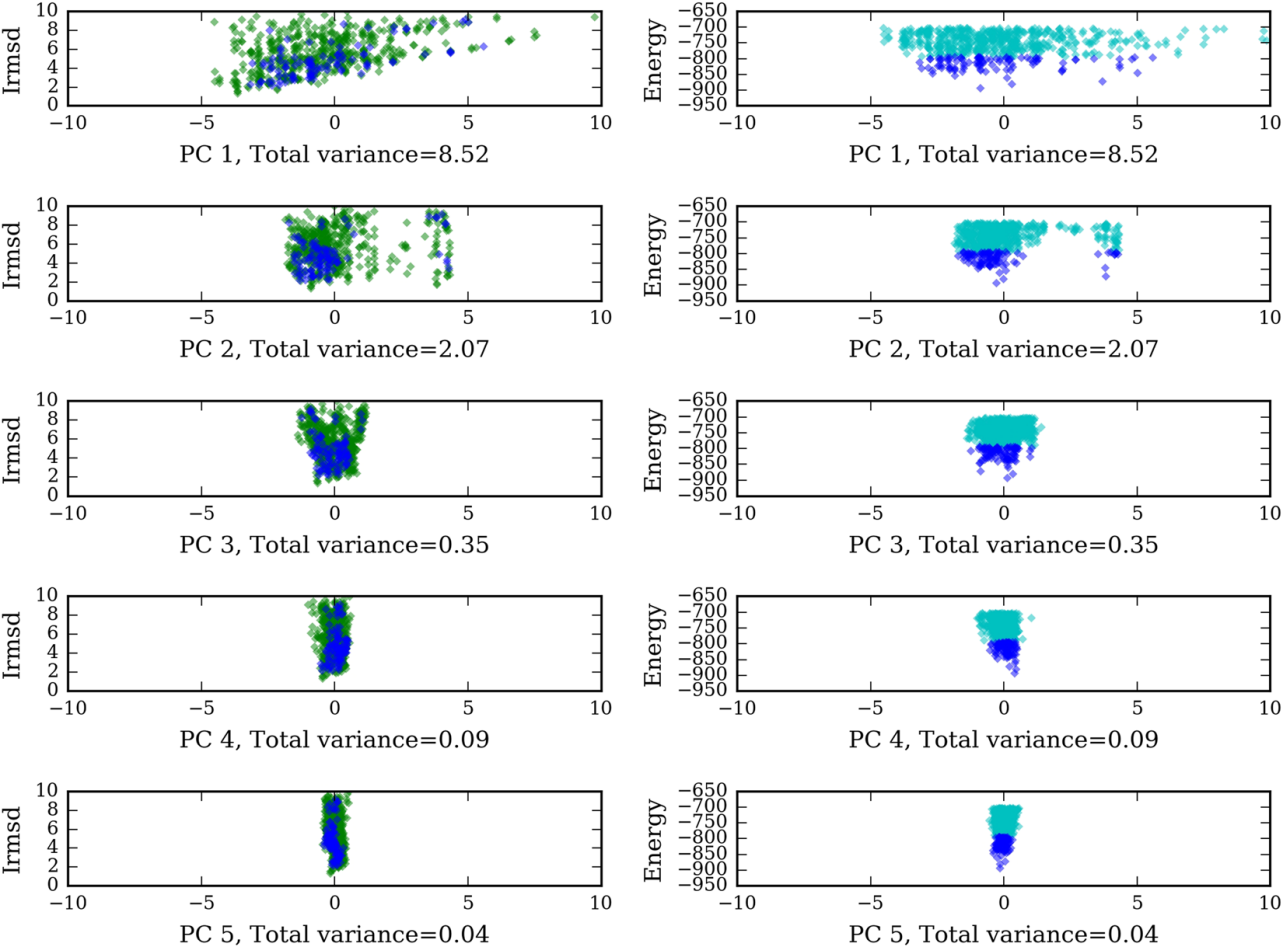
The near-native energy landscape of the 2YVJ complex using ClusPro conformations.

Realizing this, the original SDU^18,19^ removes the center-to-center distance of receptor and ligand from the 6D parameterization of the space because this dimension does not exhibit any significant variation over the ensemble of input samples, which implies a very narrow energy funnel along this dimension. These initial attempts led us to a more fundamental re-assessment of the space in which underestimation must take place. In our previous work^21^, we discovered that the near-native cluster in protein-protein complexes exhibits reduced dimensionality, suggesting that *proteins associate along preferred pathways*, similar to sliding of a protein along DNA in the process of protein-DNA recognition. We extracted the landscape features via *Principal Component Analysis (PCA)* using two distinct energy functions, one derived from PIPER sampling^2^ and the other using RosettaDock^4^. In both cases, we found that most of the variability (more than 75%) in the cluster can be explained by 3 (and sometimes 2) eigenvectors, suggesting that the energy landscape consists of a *permissive subspace* spanned by the 2 or 3 eigenvectors with the largest eigenvalues and a *restrictive landscape* spanned by the remaining eigenvectors. Figure 1 illustrates the landscape of the 2YVJ complex. It plots the distributions of Interface RMSD (root mean square deviation of interface atoms from the native) in Å and energy values based on structures generated by PIPER along the 5 eigenvectors produced by PCA, plotted from top to bottom in decreasing corresponding eigenvalue. Dark blue diamonds indicate low energy data points used for the PCA. Notice how the variability of the data points decreases from top (very wide) to bottom (very narrow).

This behavior has a deep biophysical explanation. Docking is initially driven by a diffusive search governed by Brownian motion, which brings the two molecules close. The encounter complex can be thought of as an ensemble of conformations in which the two molecules can rotationally diffuse along each other, or participate in a series of “microcollisions” that properly align the reactive groups. The second step of association consists of conformational rearrangements leading to the native complex. While it has been generally recognized that association proceeds through a transition state, little was known of the encounter complex structures and configurations, as their populations are low, their lifetimes are short, and they are difficult to trap. In our earlier work^21^, we have used results from the application of NMR Paramagnetic Relaxation Enhancement (PRE), a technique that is extremely sensitive to the presence of lowly populated states in the fast exchange regime^22–24^. Our results indicate that the PRE profiles obtained experimentally are consistent with the presence of the encounter complexes that our landscape dimensionality analysis revealed.

In this paper we use this insight to propose a new *stochastic global optimization* method we call *Subspace Semi-Definite programming-based Underestimation (SSDU)*. SSDU is based on SDU with all the generalizations we introduced earlier. The most fundamental difference however, is that underestimation takes place only in the permissive conformational subspace found by PCA. This has the effect of avoiding high-energy barriers and evaluating the energy function only at non-singular points. Since the (typically) 3D permissive subspace contains encounter complexes, the *sequence of permissive subspaces* SSDU generates amounts to a characterization of a *smooth preferred association pathway*. Put differently, these subspaces correspond to a decreasing sequence of *energy plateaux* paving a smoother way of descending to the native state.

The remainder of the paper is organized as follows. We start by presenting the SSDU algorithm (Methods). The computational results on a large benchmark set of protein structures are presented in the “Results and Discussion” Section. We conclude with some final remarks.

### Notation

Vectors will be denoted using lower case bold letters and matrices by upper case bold letters. For economy of space we write ${\bf{v}}=({v}_{1},\ldots ,{v}_{n})$ for ${\bf{v}}\in {{\mathbb{R}}}^{n}$. Prime denotes transpose. For a matrix **P**, ${\bf{P}}\,\succcurlyeq \,0$ indicates positive semi-definiteness.

## Methods

### Dimensionality Reduction

A receptor-ligand conformation can be parameterized by a 6D vector ***ψ*** = (***ρ***, ***y***), where ${\boldsymbol{\rho }}=(r,a,b)\in {{\mathbb{R}}}^{3}$ represents the translation vector from ligand center to receptor center and ${\bf{y}}=({y}_{1},{y}_{2},{y}_{3})\in {{\mathbb{R}}}^{3}$ is a parameterization of the rotation of the ligand with respect to the three axes. The space of conformations ***ψ*** is a nonlinear manifold and the parameterization of the rotations corresponds to a projection from a (flat) tangent space (in which ***y*** is defined) to the manifold itself, projecting straight lines on the tangent space map onto geodesics of the manifold. We refer the reader to the Supplement and related papers^25,26^ for a more detailed discussion of these spaces.

In the translation vector ***ρ***, *r* is the length of the vector and *a*,*b* indicate the so called exponential coordinates of the azimuth and zenith angles of ***ρ*** (see Supplement). Let us denote by $f:{{\mathbb{R}}}^{6}\to {\mathbb{R}}$ the energy function of a conformation parameterized by ***ψ*** = (*r*, *a*, *b*, *y*_1_, *y*_2_, *y*_3_).

As we mentioned earlier, in low-energy clusters where conformations are well-packed, there is no significant variation in the center-to-center distance *r* between a ligand and the receptor, and this variable can be easily optimized separately once all other variables are determined. Thus, we remove *r* from ***ψ*** and minimize *f* with respect to the remaining variables ${\bf{x}}=(a,b,{y}_{1},{y}_{2},{y}_{3})\in {{\mathbb{R}}}^{5}$.

We already discussed in the previous section that the region of the space in the neighborhood of the native state is composed of high energy barriers that prevent the ligand to move in one or two directions^21^, giving rise to a *restrictive subspace* spanned by these directions. Orthogonal to the restrictive subspace we have a *permissive subspace* where the energy is much smoother. To identify the restrictive and permissive subspaces, we apply PCA and convert the 5D parameterization of the conformational space (***x***) into linearly uncorrelated variables called *principal components* using an orthogonal transformation. This transformation seeks to find a set of principal components with the following property: the first principal component accounts for the largest possible variability in the data, and each succeeding component has the highest variance amongst all possible components which are orthogonal to the preceding components.

Suppose now we have obtained a sample of *K* local minima of *f* in the ***x***-space together with the their corresponding energy values: (***x***^(*i*)^, *f*^(*i*)^ = *f*(***x***^(*i*)^)), *i* = 1, …, *K*. We perform PCA and let ***z***^(*i*)^ be the *i*th sample point (local minimum) expressed in the basis of the principal coordinates. Our earlier work^21^ shows that in most protein-protein complexes, the first 3 PCA eigenvalues are significantly larger than the other 2 eigenvalues. Thus, we can take the first 3 principal coordinates {*z*_1_, *z*_2_, *z*_3_} to form the permissive subspace, while the remaining 2 coordinates {*z*_4_, *z*_5_} form the restrictive subspace we wish to eliminate. We denote the new coordinates of the *i* th sample point in the 3D permissive subspace by ${{\varphi }}^{(i)}=({z}_{1}^{(i)},{z}_{2}^{(i)},{z}_{3}^{(i)})\in {{\mathbb{R}}}^{3}\mathrm{.}$

Next, we aim at minimizing the energy function *f* by constructing a semidefinite underestimator over the samples ***ϕ***^(*i*)^, *i* = 1, …, *K*, in the permissive landscape.

### Underestimation

As discussed in the previous section, our method is based on finding convex underestimators which can be regarded as an approximation of the envelope spanned by the local minima of the binding energy function. In an effective underestimation, the minimum of the convex underestimator will be an approximation of the global minimum of the funnel-like binding energy function. Therefore, we can bias further sampling towards the underestimator’s minimum. Below, we first explain how the convex underestimator can be calculated, then, in the next subsection, we focus on how to bias sampling towards to the underestimator’s minimum point.

Following our earlier preliminary work^20^, we consider the class of general convex polynomial underestimators. Let *U*(***ϕ***) be a degree 2*d* polynomial and ${\varphi }\in {{\mathbb{R}}}^{n}$, where *n* = 3 in the case of seeking an underestimation in the 3D permissive subspace. In general, it is hard to show whether a general polynomial function is convex or not (except for the special case of quadratic underestimators where 2*d* = 2). It has been shown that even verifying the convexity of a degree-4 polynomial is an intractable problem^27^.

Instead, we will use a computationally tractable relaxation for convexity, called *SOS-convexity*^28^. The main idea is to verify whether a quadratic function constructed from the Hessian matrix of *U*(⋅) (the matrix of 2nd partial derivatives) is a Sum-of-Squares (see Supplement). We can then formulate the problem of finding a convex polynomial underestimator of the sample points (***ϕ***^(*i*)^, *i* = 1, …, *K*) as the following problem:

1 $$\begin{array}{ll}\mathop{{\rm{\min }}}\limits_{U(\cdot )} & \sum _{i=1}^{K}[{f}^{(i)}-U({{\varphi }}^{(i)})]\\ {\rm{s}}{\rm{.t}}{\rm{.}} & {f}^{(i)}\ge U({{\varphi }}^{(i)}),\,\,\forall i,\\  & U(\cdot )\,{\rm{is}}\,\mathrm{SOS} \mbox{-} \mathrm{convex},\end{array}$$

where the optimization is over the coefficients of the polynomial *U*(⋅). This problem can be reformulated as a standard semi-definite programming (SDP) problem (see Supplement). We use the CSDP solver^29^ to solve this SDP problem. Solving (1) provides us with the optimal coefficients of the polynomial convex function *U*(***ϕ***) that can be regarded as a tight underestimator of the *K* local minima (***ϕ***^(*i*)^, *i* = 1, …, *K*).

### Sampling

Let ${{\varphi }}^{\ast }\in {{\mathbb{R}}}^{3}$ be the global minimum of the convex underestimator obtained from the solution of (1). We will use it to sample more conformations in the vicinity of ϕ^*^. If the underestimation step succeeds in capturing the shape of the free energy function, then the sampling step will help us generate more conformations in the vicinity of the global minimum of the energy function.

First, we generate $\bar{K}$ random samples ${{\bf{s}}}^{(l)}\in {{\mathbb{R}}}^{5}$, $l=\mathrm{1,}\,\ldots ,\,\bar{K}$, where each random dimension ${s}_{i}^{(l)}$ has a uniform distribution in the range of (−0.5*βσ*_*i*_, 0.5*βσ*_*i*_), *i* = 1, …, 5, where *β* is a constant and *σ*_*i*_ is the *i* th PCA eigenvalue (*i* th diagonal element of **Σ** in Eq. (S.1) of the Supplement), where *σ*_1_ ≥ … ≥ *σ*_5_. Then, we construct the 5D global minimum z^*^ by appending an approximation of ${z}_{4}^{\ast }$, ${z}_{5}^{\ast }$ to ϕ^*^. As discussed earlier, the last two principal coordinates *z*_4_, *z*_5_ have small variation over the samples; therefore we can consider their sample mean as a good approximation, i.e., ${z}_{i}^{\ast }=\mathrm{(1/}K){\sum }_{j=1}^{K}{z}_{i}^{(j)}$, *i* = 4, 5, and set ${{\bf{z}}}^{\ast }=({{\varphi }}^{\ast },{z}_{4}^{\ast },{z}_{5}^{\ast })$.

Next, we generate the new sample points in the vicinity of the underestimator’s global minimum by randomly perturbing z^*^ along each principal coordinate by ***s***^(*l*)^. We transform these new sample points from the principal coordinates to the original coordinates, obtaining ${\tilde{{\bf{x}}}}^{(l)}$. (Specifically, ${\tilde{{\bf{x}}}}^{(l)}={\bf{W}}({{\bf{z}}}^{\ast }+{{\bf{s}}}^{(l)})+\overline{{\bf{x}}}$ where ***W*** is the matrix defined by Eq. (S.1) of the Supplement and $\overline{{\bf{x}}}$ is the mean of the *K* local minima expressed as vectors in the ***x***-space).

The sampling range of random samples ***s***^(*l*)^ at each dimension *i* is proportional to the variance *σ*_*i*_ to guarantee an effective coverage of the conformational space which preserves the sample distribution. Furthermore, in order to construct the 6D conformational parameterization of these generated sample points, we need to append the sample mean of the center-to-center distance *r*, i.e., $\bar{r}=\mathrm{(1/}K){\sum }_{i=1}^{K}{r}^{(i)}$, which results in the new sample conformation ${\tilde{{\boldsymbol{\psi }}}}^{(l)}=(\bar{r},{\tilde{{\bf{x}}}}^{(l)})\in {{\mathbb{R}}}^{6}$.

### Clustering and Outlier Elimination

As we have discussed in the Introduction, the input conformations we wish to refine may span several energy funnels. To separate these funnels before underestimation, we perform *clustering and outlier elimination*. The idea is simply to cluster the input conformations with respect to a distance measure (we use the Euclidean distance). To that end, we employ a *density-based* clustering method called *Density-Based Spatial Clustering of Applications with Noise (DBSCAN)*^30^. Given a set of sample points in the conformational space, DBSCAN groups the points which are closely packed together in a dense region and eliminates the outlier points sitting in the low-density regions. In this scheme, the dense regions are defined as the *clusters*, which are separated by the low-density regions. DBSCAN requires two input parameters: (*i*) *ε*, the distance threshold which is defined as the maximum distance of two sample points to be considered as neighbors, and (*ii*) *N*_*min*_, the minimum number of points required to form a cluster. The second parameter *N*_*min*_ ensures that all clusters found by DBSCAN will contain at least *N*_*min*_ points, and the algorithm will automatically eliminate outliers located in low-density regions.

In the case of having multiple local minima in the neighborhood of the native structure, the clustering phase will tend to group the conformations around each local minimum in a separate cluster. In the sequel, we explain how we use these clusters to handle the situations in which most of the underestimation-based refinement methods with a single underestimator^18–20^ may fail to locate the global minimum of the energy function in the near-native region.

### SSDU Algorithm

**Figure 2:**
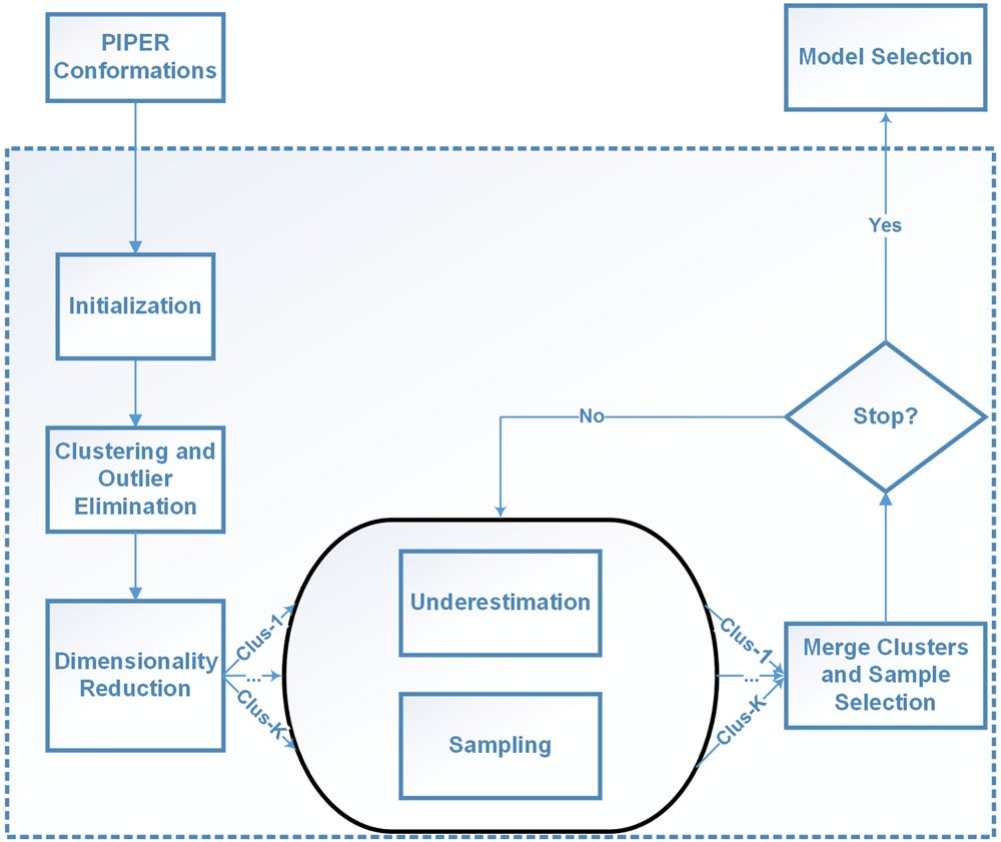
The flowchart of the SSDU procedure.

We have now described all key steps of the SSDU algorithm. The entire algorithm is outlined below in Algorithm 3. We note that the algorithm explores separately the potential multiple sub-clusters discovered by DBSCAN. Using the sampling approach we outlined, we sample *K* conformations in each sub-cluster. We then merge all these conformations and pick the lowest energy conformations. We can iterate over the steps of SSDU until meeting the stopping criteria. The retained conformations can be regarded as the SSDU outputs. Figure 2 shows a flowchart of the SSDU procedure demonstrating the process of refining the initial PIPER sample conformations to produce the ensemble of refined structures.

**Algorithm 1:**
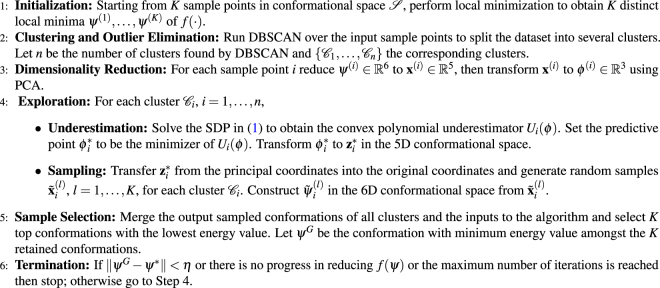
SSDU Algorithm.

### Local Minimization

All the presented sampling approaches use a common local minimization subroutine. Its main role is to account for flexibility of side chains during the search. We have explored and optimized this protocol in our previous work^31^. It consists of the following steps. We first run a *side-chain positioning (SCP)* algorithm^31,32^ that solves a relaxed formulation of a combinatorial optimization problem in order to repack the amino acid residues at the interface of the receptor-ligand complex. Then we run a *rigid-body energy minimization* algorithm^25^ which locally minimizes the position and orientation of the ligand with respect to the receptor.

### Energy Function

Our choice of energy function is a high-accuracy docking energy potential that can be calculated as a weighted sum of a number of force-field and knowledge-based energy terms^4,33,34^. Following our earlier work^5,31^, we consider the following energy terms to find the interaction free energy value:

$$E={w}_{VDW}{E}_{VDW}+{w}_{SOL}{E}_{SOL}+{w}_{COUL}{E}_{COUL}+{w}_{HB}{E}_{HB}+{w}_{DARS}{E}_{DARS}+{w}_{RP}{E}_{RP},$$

where *E*_*VDW*_ is the Lennard-Jones potential, *E*_*SOL*_ is an implicit solvation term^35^, *E*_*COUL*_ is the Coulomb potential, *E*_*HB*_ is a knowledge-based hydrogen bonding term^36^, and *E*_*DARS*_ is a structure-based intermolecular potential that is derived from the non-redundant database of native protein-protein complexes which uses a novel *DARS (Decoys as Reference State)*^37^ reference set. The last term, *E*_*RP*_, is a statistical energy term associated with a set of rotamers selected from the backbone-dependent rotamer library^38^. The weight set of the energy function is adopted according to the selections in Gray *et al*.^4^.

### Validation Dataset and Input Preparation

We validated our algorithm on a comprehensive benchmark of 230 protein complexes consisting of *Enzymes*, *Antibodies and Other types*^39^.

Other types of complexes exhibit multiple deep funnels in the vicinity of the native structure which makes them particularly difficult cases for protein docking refinement, whereas enzyme interactions are usually driven by shape complementarity, making them relatively easier cases. In fact, considering a wide spectrum of docking test cases in terms of difficulty, enables us to examine the performance gain compared to the ClusPro server in different scenarios. Moreover, other types of complexes present an opportunity to evaluate the effect of the density based clustering component built into SSDU where fitting multiple underestimators seems inevitable. Input preparation consists of two steps: (1) running global FFT sampling using PIPER; and (2) filtering the conformations to retain the top 1000 and 1500 for enzymes/antibodies and other types, respectively. These top energy conformations are supplied as the input to the SSDU algorithm.

### Data availability

The complexes we considered are part of a standard docking benchmark publicly available^40^; the structures are available through the protein data bank^41^. The code we developed is available upon reasonable request to the authors and a version will soon be released at a public depository.

## Results and Discussion

In this section, we compare the SSDU-produced ensemble with the corresponding input ensemble produced by ClusPro. We use ClusPro as a baseline for comparison because it has been established to perform comparably well to other methods^42^. In fact, ClusPro has ranked first multiple times among automated servers in the rounds of the *Critical Assessment of Prediction of Interactions* (CAPRI) community-wide experiment in the years 2009, 2013 and 2016. Furthermore, we have access to the ClusPro source code and can appropriately adjust its output for the purposes of our refinement experiments. In what follows, we consider both the number of near-native conformations in each ensemble and the implications in selecting a near-native conformation out of the refined ensemble without knowing the native structure.

The results are based on the following parameter settings: *K* = 1000 indicates the number of conformations for enzymes and antibodies and *K* = 1500 for other types of complexes, provided as the input to SSDU, *ε* = 1.0 and *N*_*min*_ = 100 are the parameters used in DBSCAN (Step 2 of Alg. 3), *η* = 0.3 (Step 6 of Alg. 3), and a maximum number of iterations equal to 3 is used for SSDU termination.

### Protein Docking Refinement

**Figure 3:**
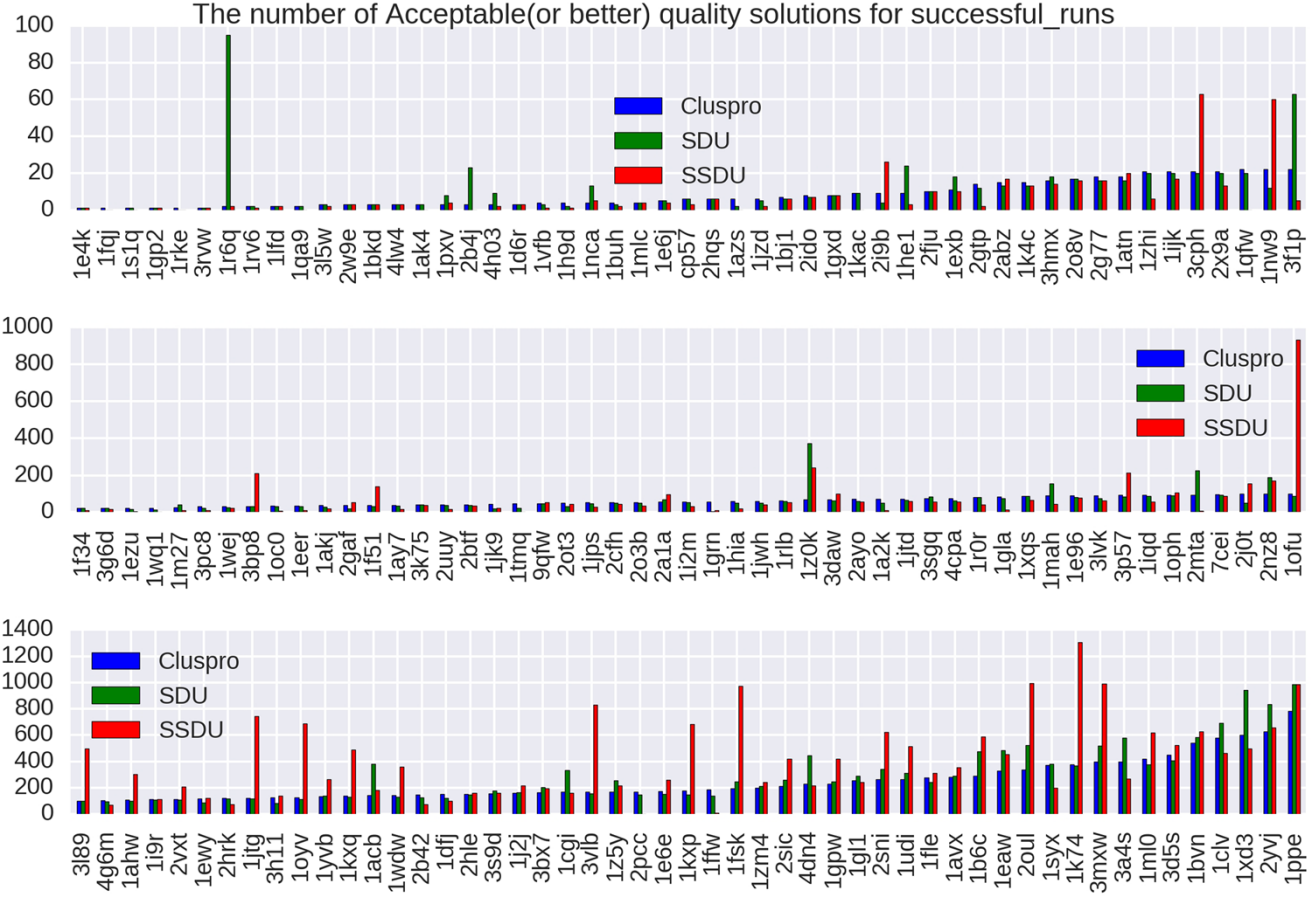
The *x*-axes of these plots list 156 out of 224 protein complexes that have either ClusPro or SSDU non-zero CAPRI Acceptable (or better) quality solutions. The complexes are sorted by the number of ClusPro counts and the *y*-axis shows the number of Acceptable (or better) quality solutions out of an ensemble of 1000 or 1500 conformations for enzymes/antibodies and other types, respectively, produced by ClusPro, or refined by SDU and SSDU.

**Figure 4:**
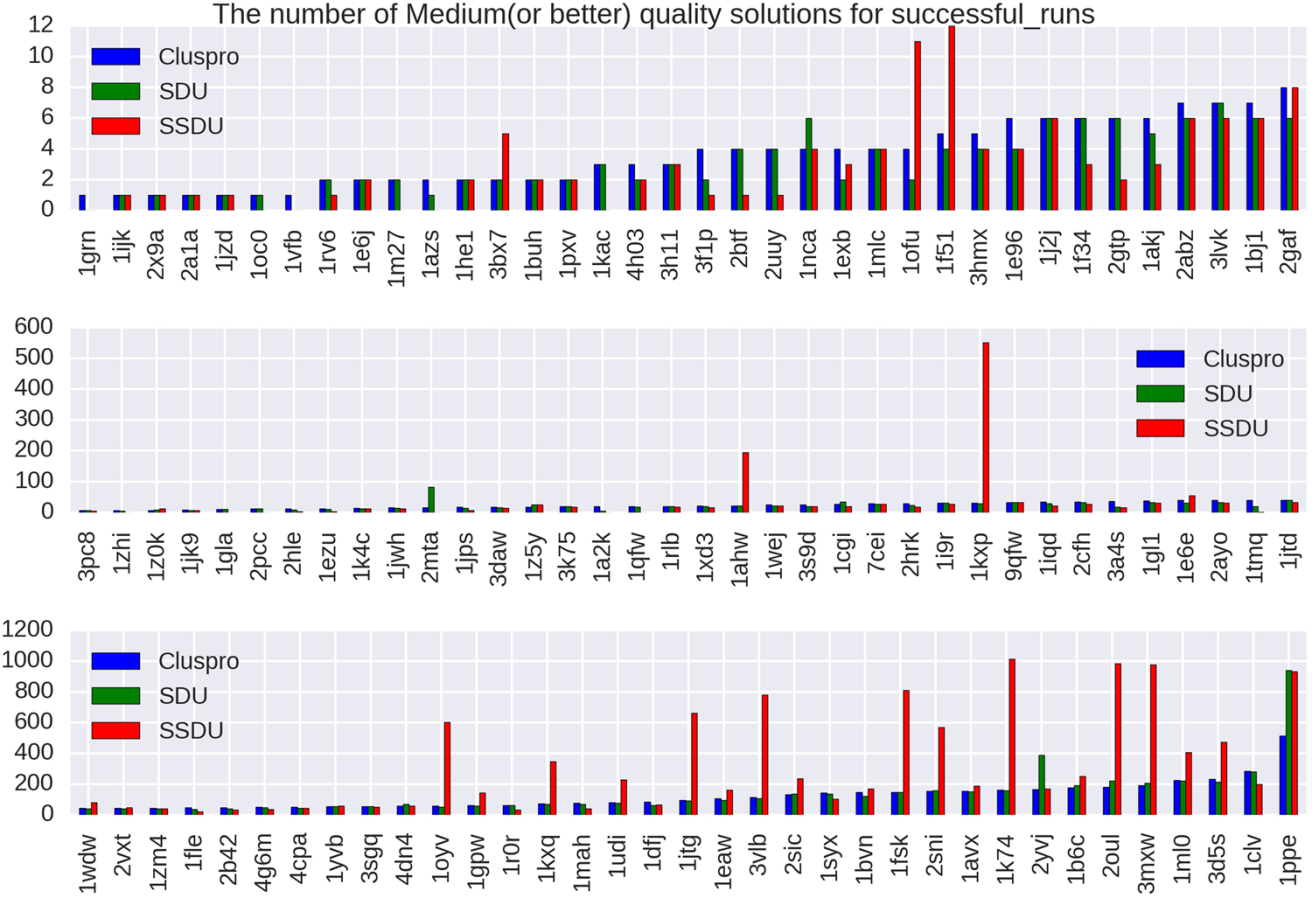
The *x*-axes of these plots list 110 out of 224 protein complexes that have either ClusPro or SSDU non-zero CAPRI Medium (or better) quality solutions. The complexes are sorted by the number of ClusPro counts and the *y*-axis shows the number of Medium (or better) quality solutions out of an ensemble of 1000 or 1500 conformations for enzymes/antibodies and other types, respectively, produced by ClusPro, or refined by SDU and SSDU.

**Figure 5:**
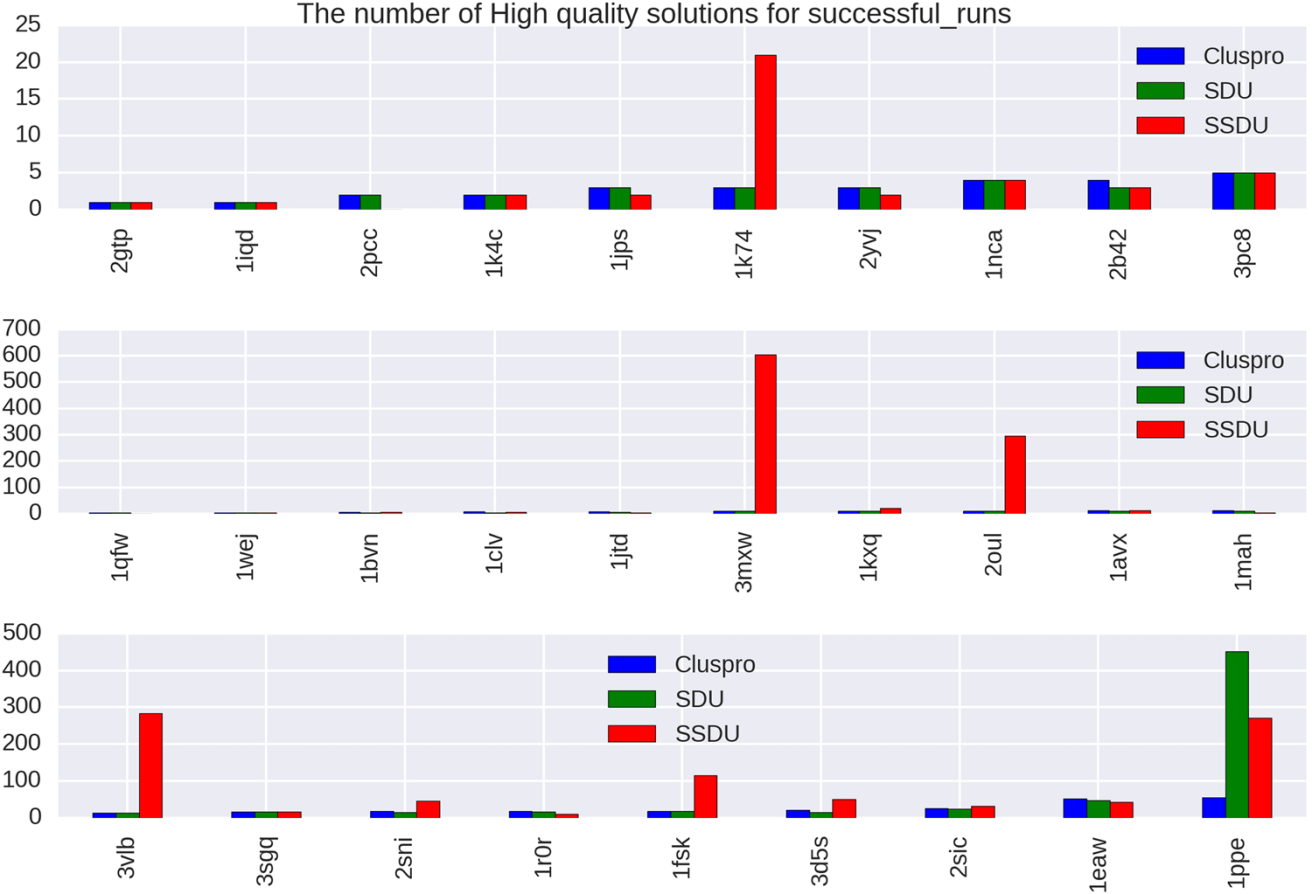
The *x*-axes of these plots list 29 out of 224 protein complexes that have either ClusPro or SSDU non-zero CAPRI High quality solutions. The complexes are sorted by the number of ClusPro counts and the *y*-axis shows the number of High quality solutions out of an ensemble of 1000 or 1500 conformations for enzymes/antibodies and other types, respectively, produced by ClusPro, or refined by SDU and SSDU.

To show the impact of the SSDU algorithm, we provide three different plots (Figs 3, 4 and 5) showing the number of Acceptable (or better), Medium (or better) and High quality solutions before and after SSDU. The classification of the quality of the solutions is based on metrics adopted in the CAPRI experiments^43^. These metrics are: interface RMSD (iRMSD), backbone RMSD (LRMSD) and the number of native contacts preserved (Fnat). To classify a conformation using these metrics, the program DockQ was used^44^. DockQ combines normalized values of iRMSD, LRMSD and Fnat to generate a continuous score in the range [0,1]; the higher the score, the better the quality of a solution. Specifically, a conformation of a protein complex is classified into four categories: Incorrect, Acceptable, Medium or High based on its DockQ score. Moreover, in addition to SSDU and ClusPro, the quality of the solutions produced by the SDU algorithm^19^ is presented as well in order to measure the performance boost from the innovations we have introduced in this paper.

We note that *the unbound protein structures* were used to generate the input to the SSDU/SDU algorithms. The use of unbound structures is important since we want to assess docking performance in the absence of any knowledge about the native conformation. As we mentioned earlier, the inputs to SSDU/SDU are the top 1000 and 1500 energy conformations from ClusPro for enzymes/antibodies and other types, respectively. The output of SSDU/SDU has the same number of conformations as the input and contains a mixture of conformations from the input and SSDU/SDU re-sampled conformations. Specifically, the re-sampled conformations from SSDU/SDU are merged with the input conformations and then subjected to energy filtering to retain the same number of lowest energy conformations as the input. For example, if the input has 1000 conformations and SSDU density-based clustering discovers three clusters, the number of conformations after merging them with the input will be 4000 (1000 per cluster and 1000 from the input), from which the 1000 lowest energy conformations are selected as the SSDU output.

We also note that we report results on 224 out of 230 complexes in the benchmark^39^. The 6 removed complexes are 4GAM, 4GXU, 2H7V, 4FQI, 1DE4, 1N2C. These complexes were removed because one of the programs we use failed to produce a score/solution for many conformations (DockQ for the first three, SSDU for the fourth, and SDU for the last two).

As it is apparent from Figs 3, 4 and 5, SSDU substantially increases the number of acceptable or better quality solutions. The amount of improvement by SSDU compared to SDU and ClusPro is reported in Table 1. The average improvement is determined by calculating the percentage improvement for each protein complex and averaging over different complexes in the benchmark, whereas the total improvement is the percentage improvement when the number of near-native hits are aggregated over all the complexes in the benchmark.

**Table 1:** Percentage improvement of Acceptable (or better), Medium (or better) and High quality solutions by SSDU versus SDU and ClusPro for a benchmark of 224 complexes. Note that for each of the entries in the table, complexes with zero number of solutions for both ClusPro and SDU/SSDU are removed.

Benchmark		SSDU vs. ClusPro	SSDU vs. SDU
Acceptable (or better)	Average	24.62%	21.31%
	Total	53.14%	30.37%
Medium (or better)	Average	53.26%	58.25%
	Total	132.69%	112.43%
High	Average	410.71%	405.88%
	Total	424.93%	157.06%

We also noticed that SSDU tends to decrease the variability of iRMSD and total energy in a near-native cluster. We provide in the Supplement an example of landscape analysis showing this effect (for the same 2YVJ complex we showed in Fig. 1).

### Post-Processing Ensemble Enrichment

We have established that SSDU generates outputs with significantly higher quality compared to the input ClusPro conformations. Next, we examine whether we can select a small number (specifically, 10) of enriched clusters from this SSDU ensemble which maintain a significant portion of the high quality conformations.

Selecting a high quality conformation remains a very challenging problem in the protein docking community. In CAPRI, participating groups test their methods in blind predictions of given target protein complexes. As mentioned before iRMSD, LRMSD and Fnat are used to categorize the predictions into Incorrect, Acceptable, Medium, and High quality. Reflecting how challenging the problem is, CAPRI allows for 10 submissions from each participating group.

ClusPro, against which we compare our results, uses clustering as a way of taking into account entropic metrics that were not included in the energy function we described earlier. Specifically, the ClusPro clustering algorithm^45^ is a greedy algorithm where, at each iteration, the structure with the largest number of neighbors is identified (two conformations are considered neighbors if their pairwise iRMSD is less than a 9 Å threshold). Then, the conformation with the highest number of neighbors is labeled a *cluster center* and along with its neighbors form a cluster and removed from the ensemble. The procedure is repeated for the remaining conformations. Overall, a maximum of 30 clusters are formed where each cluster contains at least 10 members. The collection of cluster centers produced in this manner forms a putative set of high quality conformations. ClusPro selects the centers of the 10 largest clusters as its submissions to CAPRI.

We will consider whether replacing the ClusPro ensemble with the SSDU ensemble also *enriches* the top 10 selected clusters. In this work, and because SSDU is an improved sampling method, we focus solely on the question of *cluster discrimination*, that is, selecting 10 enriched clusters. The question of *conformation discrimination*, which amounts to selecting a single representative conformation from each top cluster, is outside the scope of this paper and is left open to future work.

We form SSDU clusters by clustering the conformations in the SSDU ensemble in exactly the same way as ClusPro. We rank these clusters using a ranking method we describe in the sequel. For each complex we compare two sets of clusters. The first (ClusPro) set is formed by clustering the ClusPro produced structures and ranking the clusters in decreasing cluster size. The second (SSDU) set is formed by first refining with SSDU the ClusPro ensemble, then generating (typically 30) clusters using the ClusPro clustering algorithm, and finally ranking these clusters using the method we describe next. In each case, we compute the number of Acceptable/Medium/High quality solutions among the top 3, 5 and 10 clusters.

### Ranking the SSDU ensemble

We will next employ a machine learning approach for ranking the 30 clusters generated from the SSDU set. Some related work on using machine learning approaches, different than ours, for ranking has appeared in the literature^46,47^. We used several classification algorithms on this dataset: random forests, support vector machines with linear and radial kernels and logistic regression. *Random forests*^48^ achieved the best performance and in the remainder of this section we will focus on this classifier. To perform the classification we characterize each cluster with a set of 9 features described below:

1. The first four consist of the average energy value of the top 25%, 50%, 75% and 100% lowest energy conformations in the cluster, respectively.
2. The 5th feature is the number of conformations (size) of the cluster.
3. The last four features consist of the average RMSD between the cluster center and the top 25%, 50%, 75% and 100% conformations, respectively, in an ordered list of cluster conformations ranked in increasing RMSD from the cluster center.

We label each cluster by evaluating the DockQ score of the cluster center: if it has Acceptable quality score (or better) it is given a label of +1 (positive class); otherwise a label of −1 (negative class).

The random forest classification algorithm trains a set of unpruned de-correlated classification trees using random selection of training data and random selection of variables. It classifies a new sample by taking a majority vote of all trees, which reduces through averaging the variance of the decision. To each new sample we associate a probability of the sample belonging to the positive class as follows. The new sample is classified by each tree in the random forest and ends up in some leaf node of the tree. The percentage of training samples assigned to that leaf node which belong to the positive class is used as a surrogate of the probability that the new sample belongs to the positive class. These probabilities are then averaged over all trees in the forest to compute an overall probability that the sample belongs to the positive class. A classification decision can then be made by comparing that probability to a given threshold. Moreover, samples can be ranked using this probability.

We train random forest classifiers by randomly dividing the whole dataset into *non-overlapping* training and testing datasets, assigning 60% of the complexes in the training set and the remaining 40% to the test dataset. Because there are in general fewer clusters with a positive label, we oversampled those clusters in the training set so as to have a more balanced representation of positive and negative class clusters for training the random forest. The test dataset is not biased; it is selected at random from the entire benchmark and for each complex we select all its associated clusters. We evaluate classification performance through the Receiver Operating Characteristic (ROC) curve computed on the test set. The ROC plots the true positive rate (fraction of positive test samples correctly identified as positive) vs. the false positive rate (fraction of negative samples incorrectly identified as positive) as the threshold used for the classification decision changes. The Area Under the ROC Curve (AUC) is used as a prediction performance metric. An AUC of 1 represents perfect classification accuracy, whereas an AUC of 0.5 represents a naive random classifier which assigns samples to a class by flipping a coin.

We use the probability of a sample belonging to the positive class in order to rank (in decreasing order of the probability) the SSDU set of clusters. Similar to the ClusPro results, we count the number of Acceptable/Medium/High quality solutions among top 3, 5 and 10 clusters. Finally, we measure the improvement in the number of quality solutions in each of the three categories.

**Figure 6:**
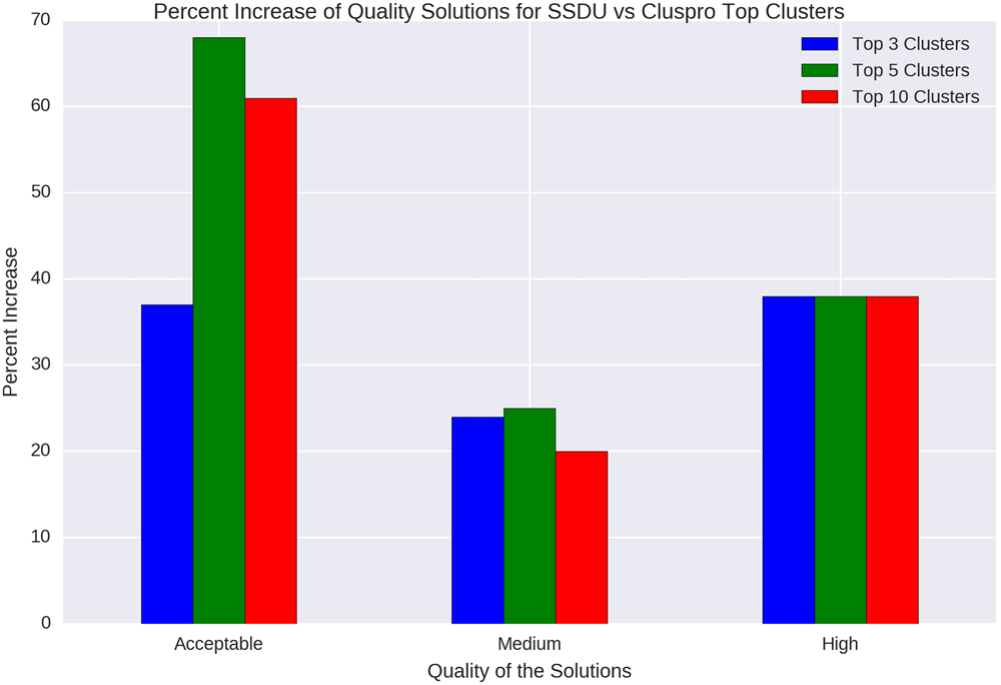
The percentage of increase in the number of Acceptable/Medium/High quality solutions among the top 3, 5 and 10 clusters achieved by SSDU over ClusPro.

As we described, we processed the SSDU cluster set using non-overlapping datasets for training and testing. We repeated training and testing 15 times, each time with a different random split of the dataset, and averaged the AUC computed on the test set (out-of-sample) over the 15 runs. This yielded an average AUC for other type of complexes equal to 0.62. This value indicates adequate classification accuracy, significantly better than random selection. Figure 6 shows the amount of improvement SSDU achieves over ClusPro for different quality categories of Acceptable/Medium/High among the top 3, 5 and 10 clusters. It is apparent from these results that SSDU can noticeably enrich the top clusters among different categories of solutions quality. For instance, SSDU can improve the density of Acceptable, Medium and High quality solutions among the top 10 clusters by 61%, 20% and 38%, respectively.

## Conclusions

We presented a new protein docking refinement protocol which is shown to effectively refine the quality of the solutions produced by first-stage global search methods like PIPER, which is implemented in the protein docking server ClusPro 2.0.

The SSDU algorithm we developed builds on our earlier SDU method^18,19^ and works by underestimating the energy function in a set of local minima generated by local minimization methods. SSDU uses the minimum of the convex underestimator it generates to concentrate further sampling in its vicinity, assuming that this minimum resides close to the basin of the energy funnel spanned by the local minima. Four innovations introduced in this work are: (*i*) the use of our landscape analysis^21^ to restrict underestimation in a lower-dimensional (typically 3D) permissive conformational subspace that avoids high-energy barriers; (*ii*) the use of density-based clustering to eliminate low-density regions and identify potential multiple high-density sub-clusters that are then separately refined by SSDU; (*iii*) the use of more flexible convex polynomial underestimators, and (*iv*) the use of a machine learning approach to effectively increase the number of Acceptable/Medium/High CAPRI quality solutions among the top clusters.

We demonstrate the effectiveness of SSDU on a comprehensive benchmark of 224 complexes containing Enzymes, Antibodies and Other Types of complexes. We show that SSDU is capable of increasing the number of quality solutions on a spectrum of different complexes in different quality categories defined by the CAPRI community-wide experiment. It was also shown that novelties introduced in this paper make SSDU superior to its predecessor SDU algorithm. Furthermore, we showed that we can further process the outputs to refine the quality of the solutions among the top clusters generated by SSDU, thereby potentially increasing the chance of picking a high quality representative from these clusters by other algorithms.

## Electronic supplementary material

Supplementary material
