# Supplementary material for: Protein docking refinement by convex underestimation in the low-dimensional subspace of encounter complexes
Source: Sci Rep. 2018 Apr 12;8:5896. doi: 10.1038/s41598-018-23982-3 (PMC5955889; doi:10.1038/s41598-018-23982-3)
Supplement: Supplementary file 1 — Supplementary material [file 41598_2018_23982_MOESM1_ESM.pdf]

Protein docking refinement by convex underestimation in the low-dimensional subspace of encounter complexes

Shahrooz Zarbafian[2], Mohammad Moghadasi[1], Athar Roshandelpoor[1], Feng Nan[1], Keyong Li[1], Pirooz Vakili[1,2], Sandor Vajda[3,*], Dima Kozakov[5,*], and Ioannis Ch. Paschalidis[1,3,4,+,*]

[1]Division of Systems Engineering, Boston University, Boston, Massachusetts, United States of America
[2]Department of Mechanical Engineering, Boston University, Boston, Massachusetts, United States of America
[3]Department of Biomedical Engineering, Boston University, Boston, Massachusetts, United States of America
[4]Department of Electrical and Computer Engineering, Boston University, Boston, Massachusetts, United States of America
[5]Department of Applied Mathematics and Statistics and Laufer Center for Physical and Quantitative Biology, Stony Brook University, Stony Brook, New York, United States of America
[+]Current address: 8 Saint Mary's St., Boston, MA 02215
[*]email address: vajda@bu.edu; midas@laufercenter.org; yannisp@bu.edu

ABSTRACT

This document includes a Supplement to the paper referenced above.

Supplementary Material

Parameterization and Dimensionality Reduction

A receptor-ligand conformation is parameterized by a 6D vector $\psi = (\rho, \mathbf{y}) \in SE(3)$, where $\rho = (r, a, b) \in \mathbb{R}^3$ is the translation vector and $\mathbf{y} = (y_1, y_2, y_3) \in \mathbb{R}^3$ specifies the exponential map coordinates for the *Special Orthogonal group* $SO(3)$ containing all rotation matrices. Here, $SE(3)$ denotes the *Special Euclidean group* — the space of rigid-body motions and the semi-direct product of $\mathbb{R}^3$ and $SO(3)$.

In the translation vector ρ, r is the length of ρ and a, b are the exponential coordinates $(a, b) = (-\omega \sin\theta, \omega\cos\theta)$, where θ is the azimuth angle between the projection of ρ on the $\rho_1\rho_2$ plane and the ρ_1 axis, and ω is the zenith angle between ρ and the ρ_3 axis.

To describe the PCA procedure, assume K local minima of f in the space of $\mathbf{x} = (a, b, y_1, y_2, y_3)$ together with the their corresponding energy values: $(\mathbf{x}^{(i)}, f^{(i)} = f(\mathbf{x}^{(i)}))$, $i = 1, \ldots, K$. Define $\mathbf{X} \in \mathbb{R}^{5\times K}$ as a matrix whose columns are of the form $\mathbf{x}^{(i)} - \bar{\mathbf{x}}$, $i = 1, \ldots, K$, where $\bar{\mathbf{x}}$ is the mean of the K local minima. We perform the eigen decomposition of $\mathbf{XX}'$

$$\mathbf{XX}' = \mathbf{W}\Sigma\mathbf{W}', \tag{S.1}$$

where $\mathbf{W}$ is a 5×5 square matrix whose ith column is the ith eigenvector of $\mathbf{XX}'$ and Σ is a diagonal matrix whose ith diagonal element is the ith corresponding eigenvalue. We use $\mathbf{z}^{(i)} = \mathbf{W}'(\mathbf{x}^{(i)} - \bar{\mathbf{x}})$ for the ith sample point transformed into the principal coordinates.

SOS Convexity

We elaborate on the notion of *SOS-convexity*[1] we used to devise convex polynomial underestimators. Let the underestimator function $U(\phi)$ be a degree $2d$ polynomial and $\phi \in \mathbb{R}^n$, where $n = 3$ in the case of seeking an underestimation in the 3D permissive subspace. Let $\mathbf{H} = \nabla^2 U(\cdot)$ be the Hessian matrix of $U(\cdot)$.

Let $\xi \in \mathbb{R}^n$ be a vector of variables, and consider $p(\phi, \xi) = \xi'\mathbf{H}(\phi)\xi$ to be a scalar polynomial of degree $2d$ with $2n$ variables (ϕ, ξ). Also, let $\mathbf{v} = (\xi_1, \ldots, \xi_n, \xi_1\phi_1, \ldots, \xi_n\phi_n^{(d-1)})$ be a vector with length $\binom{d-1+n}{n} \times n$. The following theorem[2] uses SOS-convexity as a sufficient condition for convexity.

Theorem 1 *If there exists a matrix* $\mathbf{P} \succeq 0$ *such that* $\mathbf{v}'\mathbf{P}\mathbf{v} = p(\phi, \xi) = \xi'\mathbf{H}(\phi)\xi$*, then the polynomial* $U(\cdot)$ *is convex.*

The condition in Theorem 1 is equivalent to saying that $\xi'\mathbf{H}(\phi)\xi$ is SOS (a Sum-of-Squares) in (ϕ, ξ), which suffices to ensure the convexity of $U(\cdot)$. Given this result, the optimization problem (1) in the main paper can be written by replacing the last constraint by a constraint requiring $\xi'\mathbf{H}(\phi)\xi$ is SOS in (ϕ, ξ).

We next provide an example to show how we can formulate the optimization problem (1) in the main paper as a tractable semi-definite program. Consider the special case of a degree-4 polynomial underestimator, i.e., $2d = 4$, and set $n = 3$ since we seek to underestimate in the 3D permissive subspace. In this setting the underestimator has the following form:

$$\begin{aligned}U(\phi) =& a_1 + a_2\phi_1 + a_3\phi_1^2 + a_4\phi_1^3 + a_5\phi_1^4 + a_6\phi_2 + a_7\phi_1\phi_2 + a_8\phi_1^2\phi_2 + a_9\phi_1^3\phi_2 + a_{10}\phi_2^2 + \\ & a_{11}\phi_1\phi_2^2 + a_{12}\phi_1^2\phi_2^2 + a_{13}\phi_2^3 + a_{14}\phi_1\phi_2^3 + a_{15}\phi_2^4 + a_{16}\phi_3 + a_{17}\phi_1\phi_3 + a_{18}\phi_1^2\phi_3 + \\ & a_{19}\phi_1^3\phi_3 + a_{20}\phi_2\phi_3 + a_{21}\phi_1\phi_2\phi_3 + a_{22}\phi_1^2\phi_2\phi_3 + a_{23}\phi_2^2\phi_3 + a_{24}\phi_1\phi_2^2\phi_3 + a_{25}\phi_2^3\phi_3 + \\ & a_{26}\phi_3^2 + a_{27}\phi_1\phi_3^2 + a_{28}\phi_1^2\phi_3^2 + a_{29}\phi_2\phi_3^2 + a_{30}\phi_1\phi_2\phi_3^2 + a_{31}\phi_2^2\phi_3^2 + a_{32}\phi_3^3 + a_{33}\phi_1\phi_3^3 + \\ & a_{34}\phi_2\phi_3^3 + a_{35}\phi_3^4.\end{aligned} \tag{S.2}$$

Based on Theorem 1, $\xi' H(\phi)\xi$ is SOS in (ϕ, ξ) is equivalent to $\mathbf{P} \succeq 0$ where $\mathbf{v}'\mathbf{P}\mathbf{v} = \xi' H(\phi)\xi$. Therefore, by relating the elements of $\mathbf{P}$ with coefficients of $U(\phi)$, we can reformulate (1) in the main paper as the following semi-definite problem (SDP):

$$\begin{aligned}\min_{a_1,\ldots,a_{35},\mathbf{P}} \quad & \textstyle\sum_{i=1}^K s^{(i)} \\ \text{s.t.} \quad & f^{(i)} - (a_1 + a_2\phi_1 + \cdots + a_{35}\phi_3^4) = s^{(i)}, \qquad i = 1,\ldots,K, \\ & P_{1,1} = 12a_5, \quad P_{4,4} = 2a_{12}, \quad 2P_{1,4} = 6a_9, \\ & \vdots \\ & 2P_{10,12} = 2a_{17}, \quad 2P_{11,12} = 2a_{20}, \quad P_{12,12} = 2a_{26}, \\ & \mathbf{P} \succeq 0,\ s^{(i)} \geq 0, \qquad i = 1,\ldots,K.\end{aligned} \tag{S.3}$$

To solve this SDP, we use the CSDP solver.[3] Solving (S.3) provides us with the optimal coefficients $(a_1^*, \ldots, a_{35}^*)$ of the polynomial convex function $U(\phi)$ that can be regarded as a tight underestimator of the K local minima $(\phi^{(i)}, i = 1,\ldots,K)$.

The near-native landscape after SSDU refinement

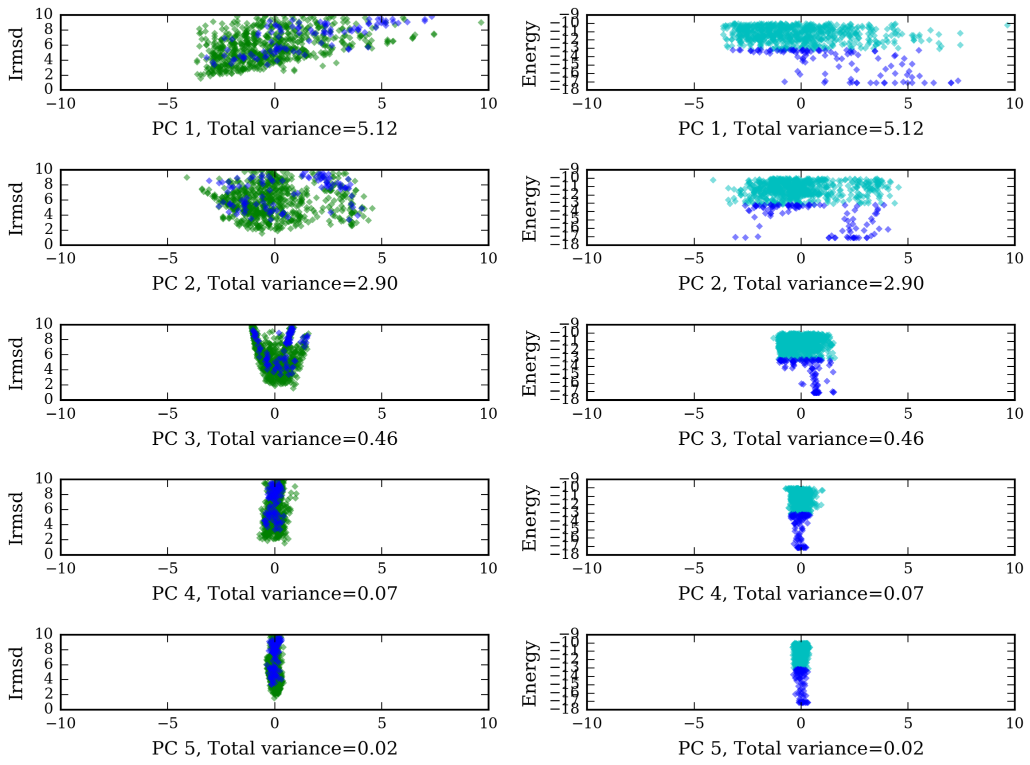

Figure A. The near-native energy landscape of the 2YVJ complex after SSDU refinement.

Fig. A shows the near-native energy landscape of the 2YVJ complex after refining the ClusPro output conformations with SSDU. It plots the iRMSD and SSDU energy distribution around the native along the five PCA eigenvectors. Notice that the largest eigenvalue decreases from 8.52 (as seen in Fig. 1 of the paper) to 5.12, a reduction of almost 40%. This implies that the SSDU refined conformations are more tightly packed around the native.

References

1. Ahmadi, A. A., Olshevsky, A., Parrilo, P. A. & Tsitsiklis, J. N. NP-hardness of deciding convexity of quartic polynomials and related problems. *CoRR* **abs/1012.1908** (2010).
2. Nan, F. *et al.* A Subspace Semi-Definite programming-based Underestimation (SSDU) method for stochastic global optimization in protein docking. In *Proceedings of the 53rd IEEE Conference on Decision and Control*, 4623–4628 (Los Angeles, California, 2014).
3. Borchers, B. CSDP, a C library for semidefinite programming. *Optim. Methods Softw.* **11**, 613–623 (1999). URL http://www.tandfonline.com/doi/pdf/10.1080/10556789908805765. DOI 10.1080/10556789908805765.
